# Enhancing Compression Level for More Efficient Compressed Sensing and Other Lessons from NMR Spectroscopy

**DOI:** 10.3390/s20051325

**Published:** 2020-02-28

**Authors:** Dariusz Gołowicz, Paweł Kasprzak, Krzysztof Kazimierczuk

**Affiliations:** 1 Centre of New Technologies, University of Warsaw, Banacha 2C, 02-097 Warsaw, Poland; d.golowicz@cent.uw.edu.pl (D.G.); pawel.kasprzak@fuw.edu.pl (P.K.); 2 Faculty of Chemistry, University of Warsaw, Pasteura 1, 02-093 Warsaw, Poland; 3 Faculty of Physics, University of Warsaw, Pasteura 5, 02-093 Warsaw, Poland

**Keywords:** compressed sensing, nuclear magnetic resonance, sparsity

## Abstract

Modern nuclear magnetic resonance spectroscopy (NMR) is based on two- and higher-dimensional experiments that allow the solving of molecular structures, i.e., determine the relative positions of single atoms very precisely. However, rich chemical information comes at the price of long data acquisition times (up to several days). This problem can be alleviated by compressed sensing (CS)—a method that revolutionized many fields of technology. It is known that CS performs the most efficiently when measured objects feature a high level of compressibility, which in the case of NMR signal means that its frequency domain representation (spectrum) has a low number of significant points. However, many NMR spectroscopists are not aware of the fact that various well-known signal acquisition procedures enhance compressibility and thus should be used prior to CS reconstruction. In this study, we discuss such procedures and show to what extent they are complementary to CS approaches. We believe that the survey will be useful not only for NMR spectroscopists but also to inspire the broader signal processing community.

## 1. Introduction

Nuclear Magnetic Resonance spectroscopy (NMR) is currently one of the most versatile techniques of chemical and physical analysis. Its range of applications is impressively broad: from analysis of small molecules structures in all states of matter [1], through characterization of complex natural mixtures [2,3], including applications to medical screening (metabolomics) [4] up to the biological studies of structure and dynamics of proteins and ribonucleic acids [5]. The introduction of Fourier transform (FT) in 1966 [6] became a cornerstone of modern NMR spectroscopy, which is based on a measurement of a free induction decay signal (FID) in a time domain. The FID is induced in a receiver coil of an NMR spectrometer by oscillating effective magnetization of nuclear magnetic moments polarized by an external magnetic field and excited by a radio frequency (RF) pulse. Importantly, the precession frequency is dependent not only on the nuclear magnetic moment and the induction of an external magnetic field but also on the electronic surrounding of a nucleus causing shielding or deshielding effect. Thus, the frequencies emitted by the sample are interesting for chemists that can deduct molecular structures from them. Formally, the precession frequency (ω) is dependent on the external magnetic field ($B_{0}$), shielding tensor (σ) and magnetic moment being a product of gyromagnetic ratio γ and spin vector *I*.

(1) $$\omega =-\gamma I\left(𝟙-\sigma \right)B_{0}$$

The FID signal s(t) typically takes a form of a sum of oscillatory decaying components:

(2) $$s\left(t\right)=\sum_{k=1}^{K}A_{k}\exp\left(i(\omega_{k}t+\phi_{k})\right)$$

The number of components *K* is equal to the number of groups of nuclei differing in resonance frequency. Each component has its amplitude ($A_{k}$) and frequency ($\omega_{k}$). The imaginary part of a frequency corresponds to a decay rate of a signal (transverse relaxation). The phase error ϕ stems from various experimental imperfections and is typically either constant or linearly dependent on the frequency ($\phi =\phi_{0}+\phi_{1}\omega$).

The concept of multidimensional data acquisition [7] opened way to measurement of *N*-dimensional FID signals that are functions of several time variables $t_{1},t_{2},\ldots ,t_{N}$. Such signals are built of products of *N* components similar to those in Equation (2):

(3) $$s\left(t_{1},t_{2},\ldots ,t_{N}\right)=\sum_{k=1}^{K}\prod_{l=1}^{N}A_{k}\exp\left(i(\omega_{k,l}t_{l}+\phi_{k,l})\right),$$

where the *k* - index in $\omega_{k,l}$ corresponds to the component’s number and *l* corresponds to the dimension of the signal (there are *N* dimensions in total, one *direct* and other *indirect*).

*N*-dimensional spectra contain useful information—not only about resonance frequencies $\omega_{k,l}$ but also about interactions exploited to trigger excitation transfer between nuclei. This allows resolving the structure of a studied molecule, i.e., to determine which nuclei are connected by single or multiple chemical bonds (transfer via spin-spin couplings), which are close in space (transfer via dipole-dipole cross-relaxation) etc. [1].

However, the acquisition of multidimensional NMR data is very time-consuming, which is cumbersome due to high costs of NMR hardware maintenance, chemical instability of some samples [8] and processes occurring in them [3]. The problem of lengthy acquisition stems from combination of three facts. First, according to the Nyquist-Shannon sampling theorem [9], the sampling rate must be at least equal to the bandwidth of a signal. Secondly, the spectral resolution is proportional to the maximum time sampled [10]. Both requirements must be fulfilled in all spectral dimensions, which means that a number of data points grows exponentially with the dimensionality of a spectrum, reaching many thousands. Finally, every sampled data point in indirect dimensions ($t_{1},t_{2}\ldots t_{N-1}$) is acquired as a separate, one-dimensional FID signal ($s(t_{N})$). The excited spin system needs to recover (or at least approach) its equilibrium state before next point is acquired, which usually takes up to a few seconds. Multiplied by several thousand indirect dimension points, this leads to even days-long NMR experiments.

Many methods have been proposed to alleviate the problem of lengthy sampling in multidimensional NMR experiments. Currently, the majority of them are based on the concept of sparse non-uniform sampling, where certain sampling points are removed from the sampling schedule and reconstructed mathematically based on various assumptions about the resulting spectrum. The assumptions may include: maximum entropy of a spectrum [11], presence of empty regions [12], minimum number of FID components [13] or minimum number of meaningful spectral points (“sparsity”) [14,15,16]. The latter assumption is a central pillar of the compressed sensing (CS) method that conquered many branches of technology and science [17], including chemical sciences [18]. The sparsest spectrum is found by minimizing the penalty function which is a sum of two terms: the first measures its accordance with the measured data and the second expressed by the $\ell_{p}$-norm (0<p≤1) of the spectrum corresponds to the spectrum sparseness. The minimum can be found by algorithms like iterative soft thresholding [14,19] (IST) or iteratively re-weighted least squares [20] (IRLS). Approaches related to famous CLEAN method also resemble $\ell_{p}$-norm minimization [21]. The concise presentation of the CS theory can be found below in Section 2.

Other techniques include the projection spectroscopy based on co-sampling of several indirect time dimensions [22], covariance spectroscopy based on non-Fourier analysis of the conventionally sampled data [23], extrapolation of such data using linear prediction [24] or attempts to remove aliasing from sampling below Nyquist rate [25]. Although their effectiveness is also based on the compressibility of the spectrum, the relationship to compressed sensing is loose and thus they are out of scope of this study.

In this work, we will examine the relation between the sampling level and compressibility of a spectrum in the context of various NMR experiments. We survey the data acquisition and signal processing tricks that enhance the compression level and show, for the first time, that it is related to the amount of data required to obtain credible spectral reconstruction with CS methods. The relation, although stems from CS theory, has never been practically verified and demonstrated to the NMR community. On the other hand, readers from outside NMR field may get inspired by the experimental tricks enhancing the compressibility and use their analogues in different contexts. Thus, we find it beneficial to share lessons from NMR spectroscopy with experts of the broadly defined signal processing field.

## 2. Theory

Compressed sensing theory is based on the concept of sparse representation of a signal and compressibility of a signal. These two features depend on a chosen basis of a signals’ vector space V. The basis $v_{1},\ldots ,v_{n}$ is usually referred to as a *dictionary*. The examples of dictionaries that on the one hand are often used by practitioners and on the other are covered by the CS theory are Fourier basis and wavelet basis [26,27]. As shown by Qu et al. [16], the former is more efficient in the case of NMR spectra. Given a dictionary $v_{1},\ldots ,v_{n}$ we say that a signal s∈V is *k*-sparse if it can be written as $\lambda_{1}v_{1}+\ldots +\lambda_{n}v_{n}$ where at least n−k$\lambda_{j}$-coefficients are equal to zero. The set of *k*-sparse signal is denoted by $\Sigma_{k}$. Please note that by simple combinatorics $\Sigma_{k}$ is a union of nk vector subspaces of V but it is not a convex subset of V.

Having the latter in mind let us formulate the central problem of CS theory. For simplicity of formulation, we shall consider the case of $V=C^{n}$ for which we fix one of the standard dictionaries (e.g., the Fourier dictionary is very useful in the NMR context). For a class of *k*-sparse signals $s=\left(s_{1},s_{2},\ldots ,s_{n}\right)\in \Sigma_{k}$ the CS theory determines the minimal number of the coordinates $s_{j}$ of the signal s required for the effective determination of the remaining ones. The *effectivity* requirement excludes the brutal search strategy, which is just checking all possible *l*-sparse signals with l≤k satisfying the measurement constraint and finding the sparsest one among them. Non-effectivity of this approach is recognized by estimating a time needed for this strategy to be implemented in case of the standard size signals, e.g., n=512 and small sparsity, e.g., k=10. The time required for solving such a problem by a brute-force would be of the order of hundreds of years (see e.g., an estimation in [28], p. 54). The CS theory provides a useful alternative for a brutal search strategy by replacing a non-convex problem (recall the non-convexity of $\Sigma_{k}$) by its convex version.

Before describing the CS approach in more details, let us note that the sparsity is undoubtedly too strong condition from the practitioner standpoint. Indeed, a signal acquired in a given experiment is contaminated with a measurement noise and the noisy part of a signal excludes its sparsity with respect to the standard dictionaries. Nevertheless, CS is still useful due to the approximate sparsity of a signal, in this work referred to as *compressibility*.

Qualitatively speaking a signal is compressible if it can be well-approximated by a sparse representation. This feature can be expressed quantitatively by means of the distance of the signal from the subset of the *k*-sparse signal $\Sigma_{k}$. The measure of this distance can be chosen in many different ways. One possibility is to use the $\ell_{p}$-norm and define

(4) $$\sigma_{k}{\left(s\right)}_{p,v_{i}}=\underset{\hat{s}\in \Sigma_{k}}{\inf}{∥s-\hat{s}∥}_{p,v_{i}}$$

where

$$∥\sum_{i}\lambda_{i}v_{i}{∥}_{p,v_{i}}={\left(\sum_{i}{\left|\lambda_{i}\right|}^{p}\right)}^{\frac{1}{p}}.$$

If $V=C^{n}$ and $\left(e_{1},e_{2},\ldots ,e_{n}\right)$ is the canonical basis of $C^{n}$ then the norm ${∥\cdot ∥}_{p,e_{i}}$ will be denoted ${∥\cdot ∥}_{p}$:

$$∥{\left(\lambda_{1},\lambda_{2},\ldots ,\lambda_{n}\right)}^{T}{∥}_{p}={\left(\sum_{i=1}^{n}{\left|\lambda_{i}\right|}^{p}\right)}^{\frac{1}{p}}.$$

Expanding a signal s in a dictionary $v_{1},\ldots ,v_{n}$

$$s=\lambda_{1}v_{1}+\ldots +\lambda_{n}v_{n}$$

the best *k*th approximation is given by keeping *k* largest components from the coordinates $\left(\lambda_{1},\lambda_{2},\ldots ,\lambda_{n}\right)$ and putting the others to zero.

Compressed sensing provides a methodology for

- highly probable exact recovery of a sparse signal based on limited information about it;
- highly probable approximately exact recovery of a compressible signal based on limited information about it.

High probability refers to the fact that recovery of the signal provided by CS method is (approximately) exact with very high probability, i.e., a wrong recovery is possible but very improbable.

To describe the convexification of CS problem let us consider the function ${∥\cdot ∥}_{0,v_{i}}$

$$V∋\sum_{i}\lambda_{i}v_{i}\mapsto \sum_{i}{\left|\lambda_{i}\right|}^{0}\in N$$

returning the number of non-zero components in the $\left(v_{i}\right)$-expansion. This is often referred to as $\ell_{0}$-norm (actually not being a norm in the mathematical sense). Fix m≤n together with a subset J⊂{1,…,n} of cardinality *m* and for every j∈J fix $s_{j}\in C^{n}$. Using $\ell_{0}$-norm one formulates the main CS problem as follows:

(5) $${\mathrm{minimize}∥x∥}_{0,v_{i}}\;\;\;\mathrm{subject}\mathrm{to}\;\;\;x_{j}=s_{j}\;\;\;\mathrm{for}\mathrm{all}\;\;\;j\in J.$$

The CS theory is based on the fundamental insight that the $\ell_{0}$-problem as formulated in Equation (5) can be replaced by its $\ell_{1}$-version

(6) $${\mathrm{minimize}∥x∥}_{1,v_{i}}\;\;\;\mathrm{subject}\mathrm{to}\;\;\;x_{j}=s_{j}\;\;\;\mathrm{for}\mathrm{all}\;\;\;j\in J$$

at least for a certain class of basis [29]. To be more precise, the solution to the problem Equation (5) coincides with the solution of Equation (6) and for this to hold there must exist sufficiently *k*-sparse solution s, and the measurement matrix must satisfy so-called uniform uncertainty principle (UUP) also known as restricted isometry property (RIP). The measurement matrix $A={\left(a_{ji}\right)}_{j\in J,i\in \{1,\ldots ,n\}}$ assigned to a basis ${\left(v_{i}\right)}_{i\in \{1,\ldots ,n\}}$ of $C^{n}$ and a fixed measurement schedule J⊂{1,…,n}, |J|=m is a matrix A with *m* rows and *n* columns such that for all j∈J

$$s_{j}=\sum_{i=1}^{n}a_{ji}\lambda_{i}.$$

We say that A satisfies uniform uncertainty principle for *k*-sparse vector if there is a constant δ>0 (sufficiently small) such that

(7) $${\left(1-\delta \right)∥\lambda ∥}_{2}\leq {∥A\lambda ∥}_{2}\leq \left(1+\delta \right){∥\lambda ∥}_{2}$$

for any $\lambda =\left(\lambda_{i}\right)\in C^{n}$ which has at least n−k zero coordinates, or in other words when $\lambda_{1}v_{1}+\ldots +\lambda_{n}v_{n}\in \Sigma_{k}$. If this condition is satisfied with sufficiently small constant δ and if the *k*-sparse solution exists then the solutions of Equation (5) and Equation (6) are equal.

Restricted isometry property for A is desired by practitioners. For specific dictionaries, this condition can be highly probable in the sense that given the sparseness level *k* and a random measurement schedule *J* of size *m*, RIP holds with high probability for sufficiently large *m*. An example of a precise criterion in the case of Fourier basis was given in [26] where the authors proved that for every ρ>0 there is a constant $D_{\rho }$ such that if

(8) $$m>D_{\rho }k\log\left(n\right)$$

then the random schedule *J* leads to the measurement matrix A which satisfies uniform uncertainty principle with probability $1-O(n^{-\rho })$. In particular, we can control the level of RIP-probability of the partial Fourier transform by choosing sufficiently large *m*, and the good news is that the number *m* of the measurement of the required signals, is linear in *k* up to the log(n) component.

The above discussion considers the idealized case of a sparse vectors’ recovery by CS method. However, the practice, in particular in NMR, immediately leads to non-sparse CS context. The first reason is the measurement error (noise) which forces the strict equality $x_{j}=s_{j}$ in Equation (6) to be replaced by an equality up to a certain error $x_{j}\approx s_{j}$. Usually ≈ is expressed by $\ell_{2}$-norm of the vector ${\left(x_{j}-s_{j}\right)}_{j\in J}$ in $C^{m}$

(9) $$∥{\left(x_{j}-s_{j}\right)}_{j\in J}{∥}_{2}={\left(\sum_{j\in J}{\left|x_{j}-s_{j}\right|}^{2}\right)}^{\frac{1}{2}}.$$

Moreover, in a certain areas of applied CS, for instance in NMR, the strict sparseness assumption must be replaced by the compressibility of signals even in the noiseless case. Indeed, the standard Lorentzian peaks present in an NMR spectrum have infinite support in frequency domain. These two facts justify the replacement of the strictly sparse CS problem Equation (6) by its relaxed approximately sparse noisy version

(10) $${\mathrm{minimize}∥x∥}_{1,v_{i}}\;\;\;\mathrm{subject}\mathrm{to}\;\;\;{∥{\left(x_{j}-s_{j}\right)}_{j\in J}∥}_{2}\leq \eta$$

where η reflects the level of the measurement errors. As proved within CS theory [30], the reconstruction algorithms are stable, i.e., for small error level and for the compressible vector s the solution of Equation (10) is close to s. To be more precise this happens if the measurement matrix has the restricted isometry property—the error of the solution of $\ell_{1}$-CS problem is measured by $\sigma_{k}{\left(x\right)}_{1,v_{i}}$, see Equation (4). In other words the *k* largest λ’s in the expansion $x=\lambda_{1}v_{1}+\ldots +\lambda_{n}v_{n}$ will be recover with high accuracy, see [29].

CS in the NMR context is often applied for the recovery of the spectra consisting of peaks with significantly different amplitudes (e.g., an NMR spectrum may contain a dominating peak whose amplitude may be even four orders of magnitude larger than the amplitudes of other peaks, see NOESY spectra). In such a case, many points contributing to the bottom part of the large (Lorentzian!) peak can be “more significant” (have higher values) than smaller peaks and thus be reconstructed in the first place. From the spectroscopist point of view, the hierarchy of importance is opposite—the small, “off-diagonal” peaks in NOESY carry the most important information. Such non-linearity of the reconstruction is also the reason the signal-to-noise ratio (SNR) in NMR spectra reconstructed with CS is not informative [31,32]. Depending on the reconstruction parameters the apparent noise level can vary from zero (high sparsity enforced) to far higher than actual (too low sparsity enforced causing incomplete removal of “NUS artifacts”). This effect can be seen in all Figures below.

To summarize this part, the concept of approximate sparsity and its relationship with the amount of data to be measured is crucial in the context of NMR experiment. Thermal noise, dynamic range of peak intensities and their linewidths, the fact that signal is complex (but only real part is of interest) create a specific framework for the application of CS to reconstruct missing points in the FID signal. Keeping this in mind, we move to the practical considerations—a bunch of *Lessons* about the effective use of CS in experimental NMR.

## 3. Results and Discussion

### 3.1. Lesson 1: Reduce the Number of Peaks

As can be seen from inequality Equation (8), the number of sampling points required for an efficient CS reconstruction (*m*) is dependent on the number of important spectral points (*k*). In the language of NMR spectroscopy, *k* is, roughly speaking, the number of points contributing to peaks. Thus, experimental techniques reducing the number of peaks to the necessary minimum not only improve spectral resolution but also allow reconstruction of the spectrum with lower *m* and shorten the experimental time.

**Figure 1:**
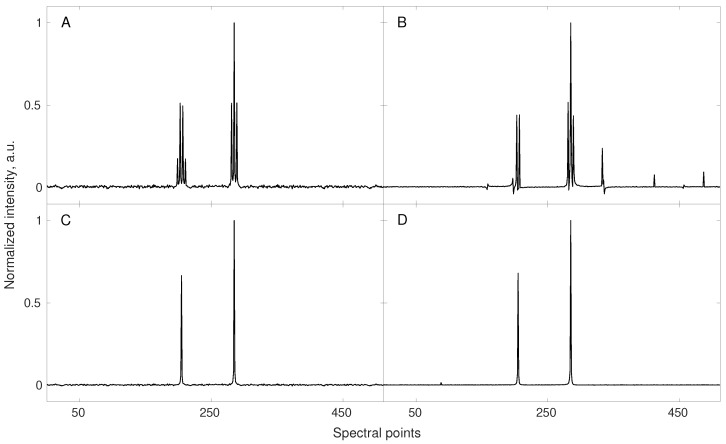
A simulation showing gains from the enhanced compressibility of the NMR spectrum obtained with pure-shift method. A conventionally sampled signal of 512 points length, containing 7 components (see corresponding triplet and quartet in spectrum (**A**)) was sub-sampled to 32 random points and reconstructed using 40 iterations of iteratively re-weighted least squares [20] algorithm (**B**). In this case, the sampling level turned out to be too low resulting in wrong reconstruction. A fully sampled (512 points) pure-shift experiment shows multiplets collapsed into the singlets (spectrum (**C**)). The corresponding reconstructed spectrum (**D**) obtained using the same sub–sampling scheme and reconstruction parameters as for (**B**) reveals to be of good quality. Reduced number of signal components (enhanced compressibility) allowed for reliable signal reconstruction using the same number of sampling points.

Many such techniques were proposed. “Pure-shift NMR” (PS-NMR) certainly belongs to the most spectacular improvements over the last decade [33,34]. The idea of PS-NMR is to remove the multiplet structure of NMR spectra by suppressing the effect of *J*-couplings i.e., interactions between nuclear spins transferred within the molecule via the nearby chemical bonds. The splittings of peaks caused by *J*-couplings can be informative but lead to the reduction of resolution and requirement of more sampling points for the proper CS reconstruction (since more peaks are present in a spectrum). With the splittings removed, less data points are required for the reconstruction (see Equation (8)). The simulation from Figure 1 shows this effect. The fact that PS-NMR naturally fits to the compressed sensing reconstruction has been discussed extensively by Aguilar and coworkers [35]. Importantly, while the couplings between nuclei of the same kind (i.e., homonuclear) can be removed using selective echo pulse sequence block in both direct and indirect dimensions of an NMR spectrum, the pseudo-random NUS is feasible only in the latter case. In the direct dimension, pure-shift experiment can be performed by sampling “chunks” of an FID signal. However, as shown in several papers [36,37,38,39] such data can also be used as an input for CS algorithms, although the RIP (cf. Equation (7)) is worse and thus more sampling points are required. Interestingly, such “burst sampling” has been reported by some authors to have also certain advantages over other sampling schemes in the indirect dimensions [40].

Besides pure-shift approach, the number of spectral peaks can also be reduced by more selective coherence transfer in correlation experiments. The selectivity is achieved by adjusting the delay time Δ in the coherence transfer block or additional coherence-selection delay. The coherence of spins coupled with *J*-constant evolves in an oscillatory manner, typically as $\sin^{n}\left(\pi J\Delta \right)$ where *n* is the number of nuclei *J*-coupled to the nucleus from which the transfer starts. The classic example is multiplicity selection [41,42] which exploits the fact that coherence transfer between interacting nuclei *A* and *X* is dependent on the multiplicity (*n*) of a spin system $AX_{n}$. For instance, CH groups can be selectively excited in a 2D ^1^H-^13^C Heteronuclear Single-Quantum Correlation spectrum (HSQC), making CH_2_ and CH_3_ peaks invisible. Similarly, one can make the transfer selective by exploiting the differences in *J* between various pairs of nuclei. In 3D HNCA, the basic experiment used to establish sequential connectivities in spectra of proteins, the excitation is initially transferred from amide hydrogens to amide nitrogens. Then, from each ^15^N nucleus the transfer may go two-fold—to α carbon of the same (*i*) and preceding (i−1) amino acid residue. This is caused by the fact that ^H^N-C_α_ coupling constants for both ways are similar (typically 11 Hz for N_i_-C_α*i*_ and 7 Hz for N_i_-C_α*i*−1_) and Δ can be set to average value in-between. However, the variants of the experiment with an exclusive transfer to one C_α_ also exist and have been used in combination with non-uniform sampling [43], also due to better compressibility of the spectrum and thus the sampling.

Another approach to achieve the reduction in a number of peaks in protein spectra was proposed by Dötsch and colleagues [44]. They modified a CBCA(CO)NH pulse sequence [45], to acquire a signal for amino acid types selected basing on topology. Only desired amino acid residues give signals in such spectra, which facilitates the analysis and allows efficient low-level non-uniform sampling [46].

Other compressibility-enhancing pulse sequence blocks allow the triggering of an exponential signal decay due to diffusion or relaxation and suppress signals selectively due to differences in the decay rate. The diffusion filter is based on the gradient echo block added to the standard NMR pulse sequence [47]. Used for mixtures of chemical compounds, it suppresses the signal from quickly diffusing smaller molecules (although it exists also in a reverse mode [48]). The somewhat opposite effect is achieved by a $T_{2}$-filter (Carr–Purcell–Meiboom–Gill sequence block [49]) which suppresses signals from nuclei with short transverse relaxation times (typically belonging to larger molecules). Diffusion-filtering and multiplicity selection, as well as their effect on the required number of sampling points, are shown in practice in Section 3.6.

### 3.2. Lesson 2: Minimize Dynamic Range

The high dynamic range of amplitudes of signal components does not constitute a significant problem for CS reconstruction in the case of signals with strictly sparse representation. The real FIDs, however, contain noise and are represented by Lorentzian peaks in the Fourier domain, with their half-width being not negligible, but proportional to the signal decay rate. The consequence is an imperfect performance of CS algorithms which are usually based on an iterative deconvolution of a point spread function (PSF) from the spectrum (for the meaning of PSF in NMR context see [50,51,52,53]). For example, one of the most classical CS algorithms, the orthogonal matching pursuit (OMP), does this by seeking for the FT basis function (an “atom”) giving the highest inner product with the FID. Then the approximation that uses only that atom is subtracted from the signal and the process is repeated. Other algorithms, like iterative (soft or hard) thresholding are based on a very similar concept [21]. The noise obviously disturbs the approximation and makes artifact removal less complete. Additionally, as mentioned in Section 2 above, the algorithm will rather tend to improve the bottom points at the sides of large Lorentzian peaks than reconstruct lower components. Thus, whenever possible, it is crucial to avoid high dynamic range of components in the spectra reconstructed with CS.

Very large and very tiny peaks are found together in spectra of mixtures of chemical compounds. It might happen, however, that some of the compounds are not interesting for the spectroscopist and can be suppressed in a spectrum. This is easy to achieve if interesting and non-interesting compounds differ significantly in the molecular size. As mentioned above, the diffusion and relaxation filters can be useful for this purpose. Unfortunately, the small and large peaks may be found even in the pure, single-compound samples. This is the case of spectra based on nuclear Overhauser effect (NOESY and ROESY). The additional difficulty arises from the fact that peaks intensities (especially those of small peaks) are the most informative parameters and thus must be reconstructed with high fidelity. This is troublesome, as series of tiny *off-diagonal* peaks are accompanied by huge (even $10^{4}\times$ larger *diagonal* peaks. To deal with this difficulty the diagonal-free NOESY experiments have been proposed [54] and shown to be particularly effective when combined with non-uniform sampling [55,56,57,58].

**Figure 2:**
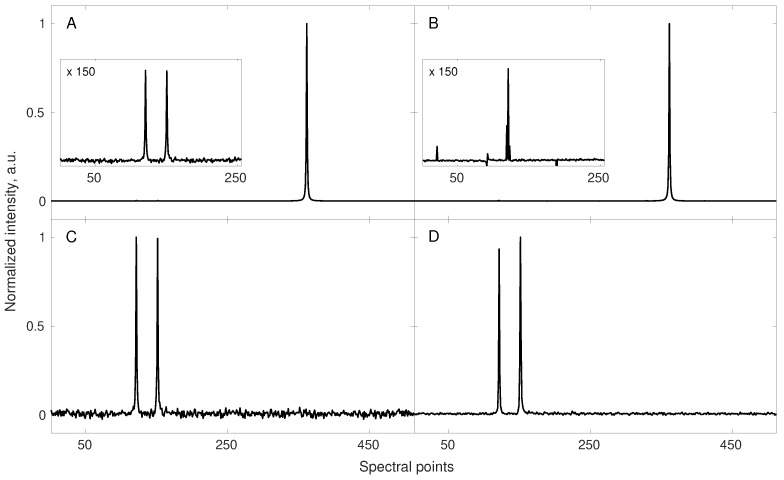
A simulation demonstrating the quality of the reconstruction of a signal built of two low amplitude components and one very high amplitude component (**A**,**B**); and of the reconstruction of the same signal with the highest amplitude component being removed (**C**,**D**). Both starting signals (their corresponding spectra—**A**,**C**) were sub-sampled to the same 42 random points (out of 512 points) and reconstructed using 40 iterations of the IRLS algorithm. The reconstruction of low amplitude signals in the presence of a high dynamic range of amplitudes was unsuccessful (**B**). The same sub-sampling level and scheme turned out to be sufficient for the fine reconstruction of the signal in the absence of a high amplitude component (**D**). The ratio of the largest peak intensity to the small ones in (**A**) was 160. Notably, the level of noise, artificially added to the FID is not reproduced in the CS reconstruction.

The Figure 2 shows this effect on a 1D cross-section along the indirect dimension of a simulated NOESY spectrum.

Notably, reducing the dynamic range of a measured object is beneficial in CS applications other than NMR. In a nice example of identification of bacterial species in a mixture by a single Sanger-sequencing reaction [59], Amir and Zuk suggested taking a square root of the data to reduce the differences between “peaks”.

### 3.3. Lesson 3: Pre-Processing

The reconstruction of missing points in NUS data from NMR experiments is one of the middle steps in the data processing workflow. This, usually, starts from the conventional procedures performed in the direct dimension: filtering, apodization, zero-filling, phasing, Fourier transform and baseline correction [60]. Importantly, before the CS reconstruction of the indirect dimension points is performed, one can apply procedures that will make the frequency representation more compressible. These include: removal of the imaginary part of a spectrum (virtual echo, VE) [61], removal of assumed modulation in the FID (virtual decoupling, VD) [62,63,64] and combination of in-phase and anti-phase (IPAP) complementary sub-signals [65,66].

**Figure 3:**
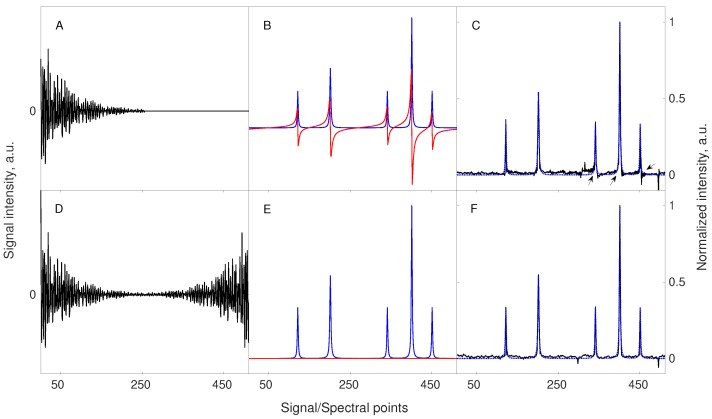
The concept of Virtual-Echo enhancing the compression level and improving the reconstruction quality. The upper panels (**A**–**C**) correspond to the signal and reconstruction without the use of VE, while the lower panels (**D**–**F**) correspond to the signal and reconstruction with VE pre-processing. A starting signal of 256 points length, containing 5 components of different amplitudes was zero-filled to 512 points (**A**) or processed accordingly to the VE method (**D**). The spectral representations of both signals, including the real (blue line) and imaginary (red line) parts, are shown in (**B**,**E**). A less sparse imaginary part of the starting signal is completely removed when VE is applied (**E**). Both signals were sub-sampled using the same sampling scheme of 48 random points. Importantly, a sampling scheme also undergoes the operation of VE in the same way as the signal. The missing points were reconstructed using 40 iterations of the CS-IRLS algorithm. The resulting spectra indicate that VE pre-processing leads to a better-quality spectrum (**F**). At this level of sampling, the spectrum reconstructed without VE pre-processing (**C**) suffers from characteristic phase distortions (see black arrows in the corresponding panel). The dotted line in panels C and F shows the real part of the fully sampled spectrum.

The first trick, virtual echo, is based on the fact that phase of the signal in the indirect dimensions (ϕ in Equation (3)) is usually known apriori, and thus the signal can be phased before the reconstruction. Thus, imaginary part of the spectrum is not needed for CS procedure and can be removed. This is beneficial, as an imaginary part of the Lorentzian line has dispersive shape and is less sparse than absorptive real part. The effect is achieved by combining FID signal with its conjugate mirror-reflected “copy” in each dimension [61]. Alternatively, a similar effect can be achieved by modifying the minimized term in Equation (10) to calculate only the real part of x [67]. The Figure 3 demonstrates the concept of VE.

The virtual decoupling is the removal of the manifestation of scalar couplings, i.e., cosine modulation of the FID, by dividing the signal by the assumed cos(πJt) function [63,64] or by equivalent modification in the algorithm [62]. The operation makes the spectrum sparser but is based on two rigorous assumptions. First, all FID components must share the same modulation (i.e., the same *J*). Secondly, division by zero must be avoided—either by regularization or by omitting zeros in the sampling schedule [68]. Contrary to virtual echo, the virtual decoupling is beneficial even in the case of fully sampled data, where no reconstruction is required. It leads to resolution and sensitivity enhancement as broad multiplets collapse into narrower and higher singlets. However, the requirement of constant *J* among all spin systems is rarely fulfilled. Typical examples are limited to adjacent carbons in 3D HNCA [64] (C_α_-C_β_ coupling) or HC-CH TOCSY spectra [63] (coupling between carbon atoms belonging to methyl and a neighboring group).

The NMR spectra are sometimes combined from subsets, as is done in case of IPAP method [65,66]. Two experiments are recorded, first providing doublets in-phase and the other anti-phase. Then, they are added which cancels the doublet components with opposite sign. In standard FT processing, it does not matter whether the addition is performed on FIDs or on spectra. For CS, however, the former is more beneficial, since it makes the spectrum more compressible (reduces the number of peaks) before the reconstruction.

The reduction of the number of components by sample preparation, sophisticated signal excitation or pre-processing is not limited to NMR and can enhance CS reconstruction in other fields. The inspiring examples can be found in CS video processing where common features of neighboring frames can be found by motion-estimation helping to enhance sparsity [69]. The sub-regions of interest in a reconstructed object can be also explicitly defined to reduce the number of significant points, as shown in the field of angiography [70].

### 3.4. Lesson 4: Match Sampling with the Decay

The vital aspect of NUS in the indirect dimensions of an NMR experiment is that the sampling schedule can be completely arbitrary. For example, its density can be modulated according to the assumed function. Besides *J*-modulation mentioned above, the useful trick is to avoid large gaps in the sampling schedule [50,71] by approaches known from other fields like jittered sampling [72] or Poisson-disk sampling [73]. Some authors show that gaps should be avoided at the beginning and at the end of a signal [74].

The oldest and most commonly applied modulation of NUS density is relaxation-matched sampling introduced by Barna et al. [75]. The gains on signal-to-noise ratio (SNR) have been analyzed in detail by other authors [31,32,76]. The reason for SNR improvement is quite simple—an FID signal decays exponentially in time, while noise level remains constant. Thus, initial sampling points have higher SNR. The measurement sensitivity aspect of the problem is simple. However, the situation becomes more complicated when analyzed from the point of view of CS theory. It can be shown that although relaxation-matching improves SNR, it worsens restricted isometry property of the measurement matrix [77]. Thus, there is certain balance between the two effects. This fact is also connected to the observation that CS works more efficiently for the interpolation of the data rather than extrapolation [78].

**Figure 4:**
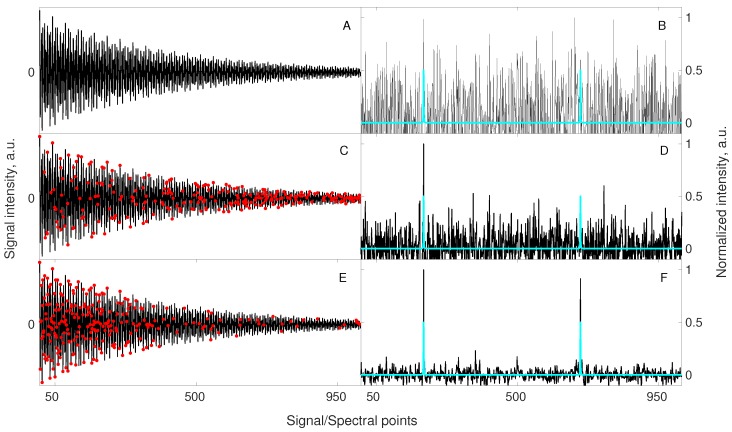
A simulation illustrating the benefit of relaxation-matched non-uniform sampling on signal reconstruction. A signal of 1024 points length containing 2 components of equal amplitudes (**A**) was artificially contaminated with a white noise such that peaks in a corresponding spectrum (**B**) were hardly visible. A blue spectrum imposed in (**B**,**D**,**F**) is obtained from the noiseless signal (**A**) to mark the correct positions of the hidden peaks (for better visualization, the peak intensities in blue spectra are normalized to half-intensity of the maximum peak in the corresponding black spectrum). The same 2-component signal was sub-sampled to 256 random points (**C**), and 256 points selected according to the relaxation-matched probability (**E**). A continuous black line in (**C**,**E**) stands for the full signal, whereas red markers correspond to sub-sampled points. Both sub-sampled sets of points were used for reconstruction using 40 iterations of the IRLS algorithm. Importantly, the sub-sampled signals (**C**,**E**) were injected into a noise being 2 times lower than for signal A. This is due to a fact that 256 points can be acquired with 4 times more scans keeping the same total experimental time, thus SNR of the acquired samples will be 2 times higher. A reconstructed spectrum (**D**), obtained from random non-uniform sampling strategy (**C**) shows no improvement, while the spectrum (**F**) obtained from a relaxation-matched non-uniform sampling strategy (**E**) indicates a significant improvement of the visibility of peaks. As described above in the text, the relaxation-matched sampling (**E**) strategy leads to better results in such cases as more samples are collected for the initial part of the signal, where SNR is higher.

Interestingly, the sensitivity benefit from relaxation-matched CS can be so strong that it can even lead to results better than fully sampled experiment acquired in the same time. The simulation in Figure 4 shows this effect.

In many applications outside NMR field, the sampling density can be adjusted “on-the-fly”, i.e., during the measurement. Such *adaptive sampling* is well established in image processing where sampling density is a function of local image variance [79,80]. In NMR spectroscopy such examples, although feasible, are still lacking.

### 3.5. Lesson 5: Non-Stationarity

**Figure 5:**
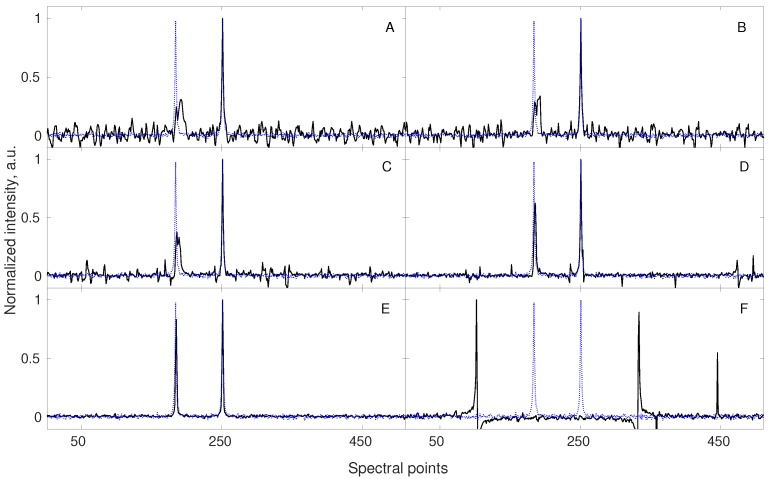
A simulation showing the benefit of applying NUS for the acquisition of a non-stationary signal (with frequency varying linearly during the experiment). A signal of 512 points length contains two components of equal amplitudes (blue dotted line in each subplot). The frequency of the component corresponding to the left peak changes by 0.05 spectral point with every NUS point acquired. The sampling levels and total frequency change are: 100% and 25.6 pts. (**A**), 75% and 19.2 pts. (**B**), 50% and 12.8 pts. (**C**), 25% and 6.4 pts. (**D**), 12.5% and 3.2 pts. (**E**), and 6.25% and 1.6 pts. (**F**). The sampling schedule is *shuffled* so the change is *not* linear in a sampled time, but in a real time of experiment. Thus, the non-stationarity leads to line broadening and additional noise-like artifacts [81,82]. Importantly, the best spectrum is obtained with 12.5 % sampling (E, far better than with full sampling A). All the NUS data sets, except of 100% NUS, were reconstructed with 40 iterations of CS–IRLS algorithm and their corresponding spectra are plotted in black.

The parameters of typical NMR signals (frequencies, amplitudes, relaxation rates) do not vary in time when measured for the stable samples. Sometimes, however, at non-stationary conditions, the frequency in some spectral dimensions varies in time [81]. Since sampling of the indirect dimensions can be arbitrary (e.g., pseudo-random), the effective frequency-time dependencies can be complicated. As demonstrated [8,81], the FID frequency varying in time leads to lineshape distortions in the case of “chronological sampling”, i.e., (t=0,Δt,2Δt,3Δt…) and noise-like artifacts in the case of “shuffled” sampling. Figure 5 shows that NUS of a non-stationary signal lead to spectral quality even better than fully sampled data. This can be explained by the fact that frequency variations within an FID, occurring, e.g., due to chemical reaction in the sample, are reduced due to shorter time needed for data collection. This means that compressed sensing should be the method of choice for samples whose state varies over the time of experiment.

In the scientific literature, one can find several interesting examples of application of NUS/CS in NMR spectroscopy for monitoring of the physical/chemical processes. The monitoring of processes involving biomolecules (e.g., proteins) is particularly interesting. In a paper by Bermel et al. [83], the NUS NMR experiment accompanied by CS reconstruction was successfully applied to monitor protein dynamics in a function of temperature. The application of NUS and CS in their work allowed to precisely track peak positions and intensities during sample heating. The reactions occurring in complex mixtures were also extensively investigated by NUS/CS NMR spectroscopy [3,84]. Usually, such mixtures require at least 2D NMR experiment to resolve important peaks in a spectrum, which makes monitoring troublesome using conventional sampling. One may also apply NUS/CS NMR spectroscopy to track different chemical reactions when a good temporal resolution and the benefits provided by 2D NMR experiments are required [85,86].

Obviously, the fact that NUS/CS experiments are quick compared to full sampling provides benefits in disciplines other than NMR. A good example has been discussed by Vasanawala et al. [87], where authors applied CS in pediatric MR imaging. Since children are rarely able to stay still during the measurement, the undersampled data is often of better quality (resolution) despite the need for reconstruction.

### 3.6. Practical Example

**Figure 6:**
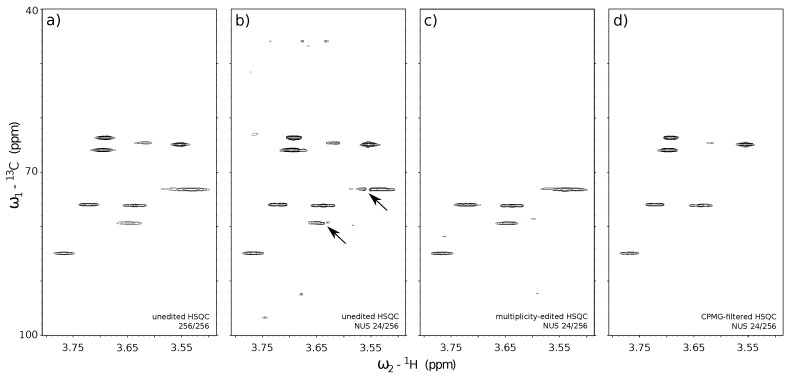
A reference unedited ^13^C HSQC spectrum with conventional sampling of 256 t_1_ (^13^C dimension) × 3348 t_2_ (^1^H dimension) points matrix (**a**) and the reconstructed spectra obtained using only 24 t_1_ sub-samples from corresponding experiments: unedited ^13^C HSQC (**b**), ^13^C HSQC with CH-only editing (**c**), ^13^C HSQC with CPMG filter (**d**). The missing data for (**b**–**d**) was reconstructed with IRLS algorithm based on CS using 40 iterations. The virtual-echo method was applied in all the reconstructions. The processing was performed using *mddnmr* software [89]. The concentration of each compound in a sample was adjusted to yield similar peak heights in the ^1^H NMR spectrum (ca. 0.6 mg/mL of sucrose, and 14.6 mg/mL of heparin).

In this section, we finally move to the experimental example of a 2D HSQC spectrum acquired with NUS and reconstructed using CS (see Figure 6). The 2D HSQC is one of the main workhorses of structural identification, acquired in huge numbers in the most of NMR labs. Thus, optimization of its speed and quality is important.

As described in Section 3.1, NMR offers a variety of different pulse sequence “blocks” that can reduce the number of observed peaks in a spectrum to the necessary minimum and hence increase its compressibility. In this section, we verify the relation between the number of peaks in a spectrum (compressibility) and the reconstruction quality. For that purpose, we acquired 3 variants of the ^13^C HSQC experiment characterized by a different number of peaks in a spectrum. The acquired 2D NMR signals for each HSQC variant were artificially sub-sampled by taking out random points from the full data in the t_1_ (^13^C) dimension and reconstructed back to the original size. The reconstructed spectra from the corresponding sub-sampled HSQC experiments are depicted in Figure 6: standard unedited ^13^C HSQC (Figure 6b), ^13^C HSQC with CH-only editing (Figure 6c) and ^13^C HSQC with CPMG filter (Figure 6d). A fully sampled, unedited ^13^C HSQC spectrum is also depicted in Figure 6a) and stands as a quality reference for the reconstructed spectra (Figure 6b–d). The ^13^C HSQC NMR experiments used in this study employ the same core HSQC pulse sequence [88], which allows observing single-quantum ^1^H–^13^C correlation signals. The use of appropriate filters (multiplicity-editing—Figure 6c and CPMG—Figure 6d) to the core HSQC sequence (Figure 6a,b) allowed us to reduce the number of components in the signal. The filters were chosen concerning the physicochemical properties of the substances being measured. The sample used for experiments was a mixture of sucrose and heparin dissolved in D_2_O. Both compounds are saccharides, but their molecular weights (MW) differ significantly, as heparin is a polysaccharide of MW in the range from 6000 up to 20,000 g/mol, while sucrose is a disaccharide of MW = 342.3 g/mol. We used this fact to suppress signals of fast-relaxing nuclei belonging to large heparin molecules by means of CPMG relaxation-filter (Figure 6d). We also used the fact that structures of sucrose and heparin consist mainly of CH and CH_2_ chemical sites, which are the source of ^1^H–^13^C single-quantum correlation signals. We employed the multiplicity-editing block to suppress signals that arise from CH_2_ chemical sites, thus, only the signals corresponding to CH sites were visible. The unedited ^13^C HSQC spectrum experiment (Figure 6a—reference and Figure 6b—reconstructed spectrum) show all the single-bond ^1^H–^13^C correlations regardless of the molecular size and type of chemical site.

The benefits of using editing and filtering “blocks” in 2D NUS NMR experiments can be found through the comparison of stacked spectra in Figure 6. The numerous ^1^H–^13^C correlation signals in the unedited ^13^C HSQC spectrum (Figure 6a) were poorly reconstructed using 24 out of 256 t_1_ sub-samples (Figure 6b). The effect is visible on heparin signals near 3.55/75.0 ppm and 3.65/80.0 ppm (marked with the black arrows in Figure 6). A reduction of the number of peaks in a spectrum allowed for more reliable reconstruction using the same 24-points sampling level for ^13^C HSQC with CH-only editing (Figure 6c) and ^13^C HSQC with CPMG filter (Figure 6d).

## 4. Conclusions

Due to high maintenance costs of high-resolution NMR spectroscopy, it is beneficial to apply sparse sampling techniques in multidimensional measurements and save the experimental time. However, since the number of sampling points required for the efficient reconstruction grows with the “complexity” of a spectrum (number of peaks and dynamic range of intensity) it is recommended to minimize it before the CS reconstruction. This can be achieved using dedicated acquisition and processing techniques. It is also noteworthy that in some cases, like strongly decaying or non-stationary signals the sparse sampling followed by the reconstruction leads to results superior to full sampling followed by Fourier transform. In this work we summarized those, often unnoticed, aspects of compressed sensing in NMR.

## Abbreviations

The following abbreviations are used in this manuscript:

CPMG	Carr–Purcell–Meiboom–Gill
CS	Compressed Sensing
DOAJ	Directory of open access journals
FID	Free Induction Decay
FT	Fourier Transform
HSQC	Heteronuclear Single-Quantum Correlation
IPAP	In-phase anti-phase
IRLS	Iteratively Re-weighted Least Squares
IST	Iterative Soft Thresholding
MDPI	Multidisciplinary Digital Publishing Institute
MW	Molecular weight
NMR	Nuclear Magnetic Resonance
NOESY	Nuclear Overhauser Effect Spectroscopy
OMP	Orthogonal matching pursuit
PS	Pure-shift (NMR)
PSF	Point spread function
RF	Radio frequency
RIP	Restricted isometry property
ROESY	Rotating Frame Overhauser Effect Spectroscopy
SNR	Signal-to-noise ratio
TOCSY	Total Correlation Spectroscopy
UUP	Uniform Uncertainty Principle
VD	Virtual Decoupling
VE	Virtual Echo

## References

[1] Simpson J.H. (2012). Organic Structure Determination Using 2-D NMR Spectroscopy.

[2] Forseth R., Schroeder F. (2012). NMR-spectroscopic analysis of mixtures: From structures to function. Curr. Opin. Chem. Biol..

[3] Dass R., Koźmiński W., Kazimierczuk K. (2015). Analysis of complex reacting mixtures by time-resolved 2D NMR. Anal. Chem..

[4] Dinges S.S., Hohm A., Vandergrift L.A., Nowak J., Habbel P., Kaltashov I.A., Cheng L.L. (2019). Cancer metabolomic markers in urine: Evidence, techniques and recommendations. Nat. Rev. Urol..

[5] Sattler M., Heidelberg E. (2004). Introduction to biomolecular NMR spectroscopy. Science.

[6] Ernst R.R., Anderson W.A. (1966). Application of Fourier Transform Spectroscopy to Magnetic Resonance. Rev. Sci. Instrum..

[7] Jeener J. (1971). AMPERE International Summer School. Basko Polje Yugoslavia.

[8] Ying J., Barnes C.A., Louis J.M., Bax A. (2019). Importance of time-ordered non-uniform sampling of multi-dimensional NMR spectra of A*β*1–42 peptide under aggregating conditions. J. Biomol. NMR.

[9] Nyquist H. (1928). Certain topics in telegraph transmission theory. Trans. Am. Inst. Electr. Eng..

[10] Szántay C. (2008). NMR and the uncertainty principle: How to and how not to interpret homogeneous line broadening and pulse nonselectivity. IV. Uncertainty. Concept. Magn. Reson. A.

[11] Mobli M., Hoch J.C. (2014). Nonuniform sampling and non-Fourier signal processing methods in multidimensional NMR. Prog. Nucl. Mag. Res. Spectrosc..

[12] Matsuki Y., Konuma T., Fujiwara T., Sugase K. (2011). Boosting protein dynamics studies using quantitative nonuniform sampling NMR spectroscopy. J. Phys. Chem. B.

[13] Qu X., Mayzel M., Cai J.F., Chen Z., Orekhov V. (2015). Accelerated NMR spectroscopy with low-rank reconstruction. Angew. Chem. Int. Ed. Engl..

[14] Kazimierczuk K., Orekhov V. (2011). Accelerated NMR spectroscopy by using compressed sensing. Angew. Chem. Int. Ed. Engl..

[15] Holland D.J., Bostock M.J., Gladden L.F., Nietlispach D. (2011). Fast multidimensional NMR spectroscopy using compressed sensing. Angew. Chem. Int. Ed. Engl..

[16] Qu X., Guo D., Cao X., Cai S., Chen Z. (2011). Reconstruction of self-sparse 2D NMR spectra from undersampled data in the indirect dimension. Sensors.

[17] Rani M., Dhok S.B., Deshmukh R.B. (2018). A Systematic Review of Compressive Sensing: Concepts, Implementations and Applications. IEEE Access.

[18] Holland D.J., Gladden L.F. (2014). Less is more: How compressed sensing is transforming metrology in chemistry. Angew. Chem. Int. Ed. Engl..

[19] Hyberts S., Milbradt A., Wagner A., Arthanari H., Wagner G. (2012). Application of iterative soft thresholding for fast reconstruction of NMR data non-uniformly sampled with multidimensional Poisson Gap scheduling. J. Biomol. NMR.

[20] Kazimierczuk K., Orekhov V.Y. (2012). A comparison of convex and non-convex compressed sensing applied to multidimensional NMR. J. Magn. Reson..

[21] Shchukina A., Kasprzak P., Dass R., Nowakowski M., Kazimierczuk K. (2017). Pitfalls in compressed sensing reconstruction and how to avoid them. J. Biomol. NMR.

[22] Coggins B.E., Venters R.A., Zhou P. (2010). Radial sampling for fast NMR: Concepts and practices over three decades. Prog. Nucl. Mag. Res. Spectrosc..

[23] Brüschweiler R., Zhang F. (2004). Covariance nuclear magnetic resonance spectroscopy. J. Chem. Phys..

[24] Koehl P. (1999). Linear prediction spectral analysis of NMR data. Prog. Nucl. Mag. Res. Spectrosc..

[25] Foroozandeh M., Jeannerat D. (2015). Reconstruction of full high-resolution HSQC using signal split in aliased spectra. Magn. Reson. Chem..

[26] Candes E.J., Romberg J., Tao T. (2006). Robust uncertainty principles: Exact signal reconstruction from highly incomplete frequency information. IEEE Trans. Inform. Theory.

[27] Donoho D.L. (2006). Compressed sensing. IEEE Trans. Inform. Theory.

[28] Foucart S., Rauhut H. (2010). A Mathematical Introduction to Compressive Sensing.

[29] Candes E.J. (2008). The restricted isometry property and its implicationsfor compressed sensing. C. R. Math..

[30] Candès E.J., Romberg J.K., Tao T. (2006). Stable signal recovery from incomplete and inaccurate measurements. Commun. Pure Appl. Math..

[31] Rovnyak D., Sarcone M., Jiang Z. (2011). Sensitivity enhancement for maximally resolved two-dimensional NMR by nonuniform sampling. Magn. Reson. Chem..

[32] Palmer M.R., Suiter C.L., Henry G.E., Rovnyak J., Hoch J.C., Polenova T., Rovnyak D. (2015). Sensitivity of nonuniform sampling NMR. J. Phys. Chem. B.

[33] Zangger K. (2015). Pure shift NMR. Prog. Nucl. Mag. Res. Spectrosc..

[34] Castañar L. (2017). Pure shift 1H NMR: What is next?. Magn. Reson. Chem..

[35] Aguilar J.A., Kenwright A.M. (2018). Compressed NMR: Combining compressive sampling and pure shift NMR techniques. Magn. Reson. Chem..

[36] Ndukwe I.E., Shchukina A., Kazimierczuk K., Butts C.P. (2016). Rapid and safe ASAP acquisition with EXACT NMR. Chem. Commun..

[37] Ndukwe I., Shchukina A., Kazimierczuk K., Cobas C., Butts C. (2016). EXtended ACquisition Time (EXACT) NMR—A Case for ’Burst’ Non-Uniform Sampling. ChemPhysChem.

[38] Ndukwe I., Shchukina A., Zorin V., Cobas C., Kazimierczuk K., Butts C. (2017). Enabling Fast Pseudo-2D NMR Spectral Acquisition for Broadband Homonuclear Decoupling: The EXACT NMR Approach. ChemPhysChem.

[39] Shchukina A., Kaźmierczak M., Kasprzak P., Davy M., Akien G.R., Butts C.P., Kazimierczuk K. (2019). Accelerated acquisition in pure-shift spectra based on prior knowledge from 1H NMR. Chem. Commun..

[40] Mobli M., Miljenović T.M. (2019). Framework for and evaluation of bursts in random sampling of multidimensional NMR experiments. J. Magn. Reson..

[41] Davis D.G. (1991). Improved multiplet editing of proton-detected, heteronuclear shift-correlation spectra. J. Magn. Reson. (1969).

[42] Kay L.E., Bax A. (1989). Separation of NH and NH2 resonances in 1H-detected heteronuclear multiple-quantum correlation spectra. J. Magn. Reson. (1969).

[43] Jaravine V.A., Zhuravleva A.V., Permi P., Ibraghimov I., Orekhov V. (2008). Hyperdimensional NMR Spectroscopy with Nonlinear Sampling. J. Am. Chem. Soc..

[44] Dötsch V., Wagner G. (1996). Editing for amino-acid type in CBCACONH experiments based on the 13 C*β*- 13 C*γ* coupling. J. Magn. Reson. Ser. B.

[45] Grzesiekt S., Bax A. (1992). Correlating Backbone Amide and Side Chain Resonances in Larger Proteins by Multiple Relayed Triple Resonance NMR. J. Am. Chem. Soc..

[46] Piai A., Gonnelli L., Felli I., Pierattelli R., Kazimierczuk K., Grudziaz K., Koźmiński W., Zawadzka-Kazimierczuk A. (2016). Amino acid recognition for automatic resonance assignment of intrinsically disordered proteins. J. Biomol. NMR.

[47] Lin M., Shapiro M.J., Wareing J.R. (1997). Diffusion-Edited NMR-Affinity NMR for Direct Observation of Molecular Interactions. J. Am. Chem. Soc..

[48] Vega-Vázquez M., Cobas J.C., Oliveira De Sousa F.F., Martin-Pastor M. (2011). A NMR reverse diffusion filter for the simplification of spectra of complex mixtures and the study of drug receptor interactions. Magn. Reson. Chem..

[49] Carr H.Y., Purcell E.M. (1954). Effects of diffusion on free precession in nuclear magnetic resonance experiments. Phys. Rev..

[50] Kazimierczuk K., Zawadzka A., Koźmiński W. (2008). Optimization of random time domain sampling in multidimensional NMR. J. Magn. Reson..

[51] Hyberts S.G., Arthanari H., Wagner G. (2012). Applications of non-uniform sampling and processing. Top. Curr. Chem..

[52] Kazimierczuk K., Orekhov V. (2015). Non-uniform sampling: Post-Fourier era of NMR data collection and processing. Magn. Reson. Chem..

[53] Zambrello M.A., Craft D.L., Hoch J.C., Rovnyak D., Schuyler A.D. (2020). The influence of the probability density function on spectral quality in nonuniformly sampled multidimensional NMR. J. Magn. Reson..

[54] Diercks T., Truffault V., Coles M., Millet O. (2010). Diagonal-free 3D/4D HN,HN-trosy-noesy-trosy. J. Am. Chem. Soc..

[55] Stanek J., Augustyniak R., Koźmiński W. (2012). Suppression of sampling artefacts in high-resolution four-dimensional NMR spectra using signal separation algorithm. J. Magn. Reson..

[56] Wen J., Zhou P., Wu J. (2012). Efficient acquisition of high-resolution 4-D diagonal-suppressed methyl-methyl NOESY for large proteins. J. Magn. Reson..

[57] Stanek J., Nowakowski M., Saxena S., Ruszczyńska-Bartnik K., Ejchart A., Koźmiński W. (2013). Selective diagonal-free 13 C, 13 C-edited aliphatic-aromatic NOESY experiment with non-uniform sampling. J. Biomol. NMR.

[58] Werner-Allen J.W., Coggins B.E., Zhou P. (2010). Fast acquisition of high resolution 4-D amide-amide NOESY with diagonal suppression, sparse sampling and FFT-CLEAN. J. Magn. Reson..

[59] Amir A., Zuk O. (2011). Bacterial community reconstruction using compressed sensing. J. Comput. Biol..

[60] Morris G.A. (2017). NMR Data Processing. Encycl. Spectrosc. Spectrom..

[61] Mayzel M., Kazimierczuk K., Orekhov V.Y. (2014). The causality principle in the reconstruction of sparse NMR spectra. Chem. Commun..

[62] Shimba N., Stern A.S., Craik C.S., Hoch J.C., Dötsch V. (2003). Elimination of 13C*α* splitting in protein NMR spectra by deconvolution with maximum entropy reconstruction. J. Am. Chem. Soc..

[63] Kerfah R., Hamelin O., Boisbouvier J., Marion D. (2015). CH3-specific NMR assignment of alanine, isoleucine, leucine and valine methyl groups in high molecular weight proteins using a single sample. J. Biomol. NMR.

[64] Robson S.A., Takeuchi K., Boeszoermenyi A., Coote P.W., Dubey A., Hyberts S., Wagner G., Arthanari H. (2018). Mixed pyruvate labeling enables backbone resonance assignment of large proteins using a single experiment. Nat. Commun..

[65] Ottiger M., Delaglio F., Bax A. (1998). Measurement of J and Dipolar Couplings from Simplified Two-Dimensional NMR Spectra. J. Magn. Reson..

[66] Andersson P., Weigelt J., Otting G. (1998). Spin-state selection filters for the measurement of heteronuclear one-bond coupling constants. J. Biomol. NMR.

[67] Stern A.S., Hoch J.C. (2015). A new approach to compressed sensing for NMR. Magn. Reson. Chem..

[68] Jaravine V., Ibraghimov I., Orekhov V.Y. (2006). Removal of a time barrier for high-resolution multidimensional NMR spectroscopy. Nat. Methods.

[69] Liu Y., Li M., Pados D.A. (2013). Motion-aware decoding of compressed-sensed video. IEEE Trans. Circuits Syst. Video Technol..

[70] Konar A.S., Aiholli S., Shashikala H.C., Babu D.R., Geethanath S. Application of Region of Interest Compressed Sensing to accelerate magnetic resonance angiography. Proceedings of the 2014 36th Annual International Conference of the IEEE Engineering in Medicine and Biology Society, EMBC 2014.

[71] Kazimierczuk K., Zawadzka A., Koźmiński W., Zhukov I. (2007). Lineshapes and artifacts in Multidimensional Fourier Transform of arbitrary sampled NMR data sets. J. Magn. Reson..

[72] Mitchell D.P. Generating antialiased images at low sampling densities. Proceedings of the 14th Annual Conference on Computer Graphics and Interactive Techniques, SIGGRAPH 1987.

[73] Lagae A., Dutré P. (2008). A comparison of methods for generating Poisson disk distributions. Comput. Graph. Forum.

[74] Hyberts S.G., Takeuchi K., Wagner G. (2010). Poisson-gap sampling and forward maximum entropy reconstruction for enhancing the resolution and sensitivity of protein NMR data. J. Am. Chem. Soc..

[75] Barna J.C., Laue E.D., Mayger M.R., Skilling J., Worrall S.J. (1987). Exponential sampling, an alternative method for sampling in two-dimensional NMR experiments. J. Magn. Reson. (1969).

[76] Paramasivam S., Suiter C.L., Hou G., Sun S., Palmer M., Hoch J.C., Rovnyak D., Polenova T. (2012). Enhanced sensitivity by nonuniform sampling enables multidimensional MAS NMR spectroscopy of protein assemblies. J. Phys. Chem. B.

[77] Kazimierczuk K., Lafon O., Lesot P. (2014). Criteria for sensitivity enhancement by compressed sensing: Practical application to anisotropic NAD 2D-NMR spectroscopy. Analyst.

[78] Hyberts S.G., Robson S.A., Wagner G. (2017). Interpolating and extrapolating with hmsIST: Seeking a t_*max*_ for optimal sensitivity, resolution and frequency accuracy. J. Biomol. NMR.

[79] Lee M.E., Redner R.A., Uselton S.P. (1985). Statistically Optimized Sampling for Distributed Ray Tracing. Comput. Graph. (ACM).

[80] Kajiya J.T. The rendering equation. Proceedings of the 13th Annual Conference on Computer Graphics and Interactive Techniques, SIGGRAPH 1986.

[81] Gołowicz D., Kasprzak P., Orekhov V., Kazimierczuk K. (2019). Fast time-resolved NMR with non-uniform sampling. Prog. Nucl. Mag. Res. Spectrosc..

[82] Dass R., Kasprzak P., Koźmiński W., Kazimierczuk K. (2016). Artifacts in time-resolved NUS: A case study of NOE build-up curves from 2D NOESY. J. Magn. Reson..

[83] Bermel W., Dass R., Neidig K.P., Kazimierczuk K. (2014). Two-Dimensional NMR Spectroscopy with Temperature-Sweep. ChemPhysChem.

[84] Dass R., Grudzia̧ż K., Ishikawa T., Nowakowski M., Dbowska R., Kazimierczuk K. (2017). Fast 2D NMR spectroscopy for in vivo monitoring of bacterial metabolism in complex mixtures. Front. Microbiol..

[85] Wu Y., D’Agostino C., Holland D.J., Gladden L.F. (2014). In situ study of reaction kinetics using compressed sensing NMR. Chem. Commun..

[86] Gołowicz D., Kazimierczuk K., Urbańczyk M., Ratajczyk T. (2019). Monitoring Hydrogenation Reactions using Benchtop 2D NMR with Extraordinary Sensitivity and Spectral Resolution. ChemistryOpen.

[87] Vasanawala S.S., Alley M.T., Hargreaves B.A., Barth R.A., Pauly J.M., Lustig M. (2010). Improved pediatric MR imaging with compressed sensing. Radiology.

[88] Kay L.E., Keifer P., Saarinen T. (1992). Pure absorption gradient enhanced heteronuclear single quantum correlation spectroscopy with improved sensitivity. J. Am. Chem. Soc..

[89] Orekhov V.Y., Jaravine V., Mayzel M., Kazimierczuk K. MddNMR—Reconstruction of NMR Spectra from NUS Signal Using MDD and CS. http://mddnmr.spektrino.com.

